# Ampicillin/Sulbactam Vs. Cefoxitin for the Treatment of Pelvic Inflammatory Disease

**DOI:** 10.1155/S1064744997000562

**Published:** 1997

**Authors:** Joseph G. Jemsek, Frank Harrison

**Affiliations:** 1 Department of Obstetrics and Gynecology Charlotte Memorial Hospital Charlotte NC USA; 2 Nalle Clinic 1918 Randolph Road Charlotte NC 28207 USA

## Abstract

*Objective:* The safety and efficacy of ampicillin plus sulbactam were compared with those of cefoxitin in the treatment of women with pelvic inflammatory disease (PID).

*Methods:* This single-site, randomized, prospective, third-party-blinded, comparative, parallel-treatment study enrolled 93 women with a diagnosis of PID. Patients were treated with either ampicillin/sulbactam (2 g/1 g, administered intravenously [IV], every 6 h) or cefoxitin (2 g, administered IV, every 6 h) for a minimum of 12 doses. Patients with cultures positive for *Chlamydia trachomatis* also received concurrent oral or IV doxycycline (100 mg twice daily). Patients with cultures negative for *C. trachomatis* received prophylactic oral doxycycline (100 mg twice daily) for 10–14 days after treatment with either ampicillin/sulbactam or cefoxitin was completed.

*Results:* Ninety-three patients were entered in the study: 47 in the ampicillin/sulbactam arm and 46 in the cefoxitin arm. All 93 patients were evaluable for safety; 61 (66%) were evaluable for efficacy. Demographic characteristics were similar for the groups. Of the 27 evaluable ampicillin/sulbactam-treated patients, 67% experienced clinical cure, 30% improved, and 4% failed treatment. Respective values for the 34 cefoxitin-treated patients were 68%, 24%, and 9% (*P* = 0.67). Pathogens were eradicated in 70% of the women given ampicillin/sulbactam vs. 56% of those who received cefoxitin (*P* = 0.64).

*Conclusions:* Overall, ampicillin/sulbactam demonstrated clinical and bacteriologic efficacy at least equivalent to that of cefoxitin in the treatment of women with acute PID. The use of ampicillin/sulbactam for this indication may avoid the complex dosing regimens associated with other treatments.


Infectious Diseases in Obstetrics and Gynecology 5:319–325 (1997)
© 1998 Wiley-Liss, Inc.

### Ampicillin/Sulbactam Vs. Cefoxitin for the Treatment of Pelvic Inflammatory Disease

Joseph G. Jemsek* and Frank Harrison
*Department of Obstetrics and Gynecology, Charlotte Memorial Hospital, Charlotte, NC*

### Abstract

*Objective:* The safety and efficacy of ampicillin plus sulbactam were compared with those of cefoxitin in the treatment of women with pelvic inflammatory disease (PID).

*Methods:* This single-site, randomized, prospective, third-party-blinded, comparative, parallel-treatment study enrolled 93 women with a diagnosis of PID. Patients were treated with either ampicillin/sulbactam (2 g/1 g, administered intravenously [IV], every 6 h) or cefoxitin (2 g, administered IV, every 6 h) for a minimum of 12 doses. Patients with cultures positive for *Chlamydia trachomatis* also received concurrent oral or IV doxycycline (100 mg twice daily). Patients with cultures negative for *C. trachomatis* received prophylactic oral doxycycline (100 mg twice daily) for 10–14 days after treatment with either ampicillin/sulbactam or cefoxitin was completed.

*Results:* Ninety-three patients were entered in the study: 47 in the ampicillin/sulbactam arm and 46 in the cefoxitin arm. All 93 patients were evaluable for safety; 61 (66%) were evaluable for efficacy. Demographic characteristics were similar for the groups. Of the 27 evaluable ampicillin/sulbactam-treated patients, 67% experienced clinical cure, 30% improved, and 4% failed treatment. Respective values for the 34 cefoxitin-treated patients were 68%, 24%, and 9% ($P = 0.67$). Pathogens were eradicated in 70% of the women given ampicillin/sulbactam vs. 56% of those who received cefoxitin ($P = 0.64$).

*Conclusions:* Overall, ampicillin/sulbactam demonstrated clinical and bacteriologic efficacy at least equivalent to that of cefoxitin in the treatment of women with acute PID. The use of ampicillin/sulbactam for this indication may avoid the complex dosing regimens associated with other treatments. Infect. Dis. Obstet. Gynecol. 5:319–325, 1997. © 1998 Wiley-Liss, Inc.

Key words
pelvic inflammatory disease; antibiotic therapy; β-lactam; β-lactamase; ampicillin/sulbactam; cefoxitin

Pelvic inflammatory disease (PID) is a common and serious complication of sexually acquired infection.[1] From 1979 to 1988, an average of nearly 190,000 women were hospitalized annually for PID, and nearly 400,000 first visits per year for PID were made to physicians' offices.[1] According to more recent estimates, PID results in approximately 2.5 million office visits and 267,000 hospitalizations each year in the United States alone.[2] Serious long-term consequences of PID, including chronic pelvic pain, infertility, ectopic pregnancy, or major pelvic or abdominal surgery, will affect one fourth of the women with this infection.[3,4]

Although *Neisseria gonorrhoeae* and *Chlamydia trachomatis* are most commonly associated with PID,[4–6] this disease may arise from both aerobic and anaerobic flora normally present in the lower genital tract.[7,8] Among causative pathogens are aerobic organisms such as *Escherichia coli*, group B streptococci, and other facultative streptococci, as well as anaerobic organisms including *Prevotella bi-*

Contract grant sponsor: Pfizer Pharmaceuticals.
*Correspondence to: Dr. Joseph G. Jemsek, Nalle Clinic, 1918 Randolph Road, Charlotte, NC 28207.

Received 27 December 1996
Accepted 28 October 1997

*vius*, other *Prevotella* species, and peptostreptococci.[7]

The polymicrobial nature of PID requires empiric therapy with a broad-spectrum antimicrobial agent or combination.[8,9] Complicating effective therapy, however, has been the development of significant antibiotic resistance[8–10] through production of penicillinase by *N. gonorrhoeae* and of β-lactamase by various other causative pathogens of PID.[8–10] Although combination therapy with gentamicin and clindamycin has been considered the "gold standard,"[8,11] in recent clinical trials[9,10,12] the combination of the β-lactam ampicillin and the β-lactamase inhibitor sulbactam has demonstrated excellent activity against pathogens associated with pelvic infections in women.[13,14]

The present study was undertaken to directly compare the efficacy and safety of ampicillin/sulbactam and cefoxitin, a semisynthetic cephamycin, in women with PID.

### SUBJECTS AND METHODS

Eligible for enrollment were females 14 years of age or older who provided informed written consent (including parental or legal guardian consent for patients <18 years of age) and who had a diagnosis of PID based on medical history, clinical laboratory findings, physical examination, and clinical signs and symptoms of infection, including abdominal, adnexal, or cervical motion tenderness. Leukocyte count ≥10,000 mm$^3$, temperature ≥100.4°F, left shift (bands ≥10%), or endocervical discharge positive for gram-negative intracellular diplococci were also required. Ultrasonography was employed, as necessary, to rule out the presence of abdominal abscess.

Before study initiation, the study protocol and statement of informed consent were reviewed and approved by the institutional review committee.

Criteria for exclusion were known hypersensitivity to cephalosporins or penicillins; need for concomitant antibiotic therapy; terminal illness with death likely within 48 h; severe underlying disease that might interfere with evaluation of the therapeutic response; successful antimicrobial therapy within 4 days prior to study entry; ongoing treatment with another investigational drug; impaired immunological function or neutropenia (neutrophil count <1,500 mm$^3$); serum creatinine >1.5 mg/dl; pregnancy; or known liver disease (liver function test ≥twice normal value).

Patients provided medical and surgical histories and underwent physical examination. Within 48 h prior to initiation of therapy, endocervical specimens were taken for aerobic culture of *N. gonorrhoeae, C. trachomatis, Mycoplasma hominis*, and *Ureaplasma urealyticum*. Uncontaminated specimens from normally sterile sites taken during culdocentesis, laparoscopy, or laparotomy were also cultured for aerobes and anaerobes. Follow-up endocervical cultures were not required, except to document treatment failure. Because PID may have a clinical presentation similar to, or may occur concurrently with, pyelonephritis, at least 2 blood cultures with specimens from separate sites were performed to aid in the differential diagnosis; follow-up cultures were obtained if bacterial growth was noted. All probable bacterial pathogens were tested for susceptibility to ampicillin, ampicillin/sulbactam, and cefoxitin.

Within 24 h of admission, patients were tested for *C. trachomatis* by the immunofluorescent antibody test. A positive result was defined as *C. trachomatis* on either this test or culture.

Pretreatment blood samples were collected for complete blood count with differential, volume, and platelet count. Prothrombin time and partial thromboplastin time were performed in patients with signs of bleeding disorders. Blood chemistry profiles included alanine and aspartate aminotransferases, serum creatinine, and blood urea nitrogen. Urinalysis was also performed. Pretreatment and follow-up radiographic and sonographic procedures were carried out at the investigator's discretion.

Patients were randomly assigned to receive either ampicillin/sulbactam (2 g/1 g) or cefoxitin (2 g) both administered intravenously (IV) every 6 h. Doxycycline, 100 mg twice daily, either oral or IV, was administered concurrently to patients with cultures positive for *C. trachomatis*. Because of the significant possibility of false negatives, patients with cultures negative for *C. trachomatis* were empirically treated with 10–14 days of oral doxycycline, 100 mg twice daily, after the study ended. A minimum of 12 doses of ampicillin/sulbactam and cefoxitin were given.

Based on the clinical judgment of the investigator, other antibiotics or non-pharmacological interventions were administered to treatment failures.

Patients were withdrawn from the study for the following reasons: obvious therapeutic failure of the study drug; primary pathogen isolated from an initial culture, or any subsequent culture taken during the treatment period, resistant to the study drug(s) and patient failure to respond to therapy within 48 h; a significant adverse event (including significant alteration in laboratory parameters); or request for withdrawal by the patient or the parent (guardian) of a minor patient.

An initial clinical evaluation was performed on the first day of therapy, at least every other day thereafter, and at the end of study drug treatment. Signs and symptoms of pelvic infection were evaluated. Temperature was recorded, and the presence or absence of abdominal abscess was noted.

One week after treatment with study drug ended, all patients underwent a follow-up assessment. An endocervical specimen was taken at this time from patients with initial *C. trachomatis* or *N. gonorrhoeae* infection to test for eradication. An additional follow-up was carried out 4–6 weeks after the end of treatment.

Whenever possible, sexual partners of patients testing positive for sexually transmitted disease (STD) species were treated. At time of discharge, patients were instructed by the treating physician to avoid vaginal sexual exposure before follow-up visits. The use of condoms was encouraged.

The clinical response to treatment was evaluated as cure: disappearance of presenting signs and symptoms by the end of therapy and at follow-up; improvement: partial alleviation of signs and symptoms by the end of therapy and at follow-up; failure: no significant effect on signs and symptoms; or indeterminate: results not evaluable or not fitting into one of the other categories.

Bacteriologic response to treatment was defined as eradication: disappearance of culturable material or elimination of the principal pathogen(s) at the end of therapy and at follow-up; partial eradication: some, but not all, multiple causative pathogens absent after completion of therapy; eradication/superinfection: elimination of the principal pathogen(s) and emergence of a different organism during, or at the end of, therapy and at follow-up; persistence of the principal pathogen at the end of therapy and at follow-up; or indeterminate: results not evaluable or not definable per protocol.

**TABLE 1. Demographic characteristics of evaluable patients[a]**

| | Ampicillin/sulbactam (N = 27) | Cefoxitin (N = 34) | *P* |
|---|---|---|---|
| Age (years) | 22.0 ± 5.25 | 23.4 ± 4.70 | 0.34 |
| Race | | | |
| White | 4 | 4 | 0.73 |
| Black | 23 | 30 | |
| Weight (lb) | 130.5 ± 24.40 | 136.3 ± 25.04 | 0.81 |
| Height (in.) | 63.8 ± 2.34 | 64.2 ± 3.43 | 0.73 |

[a]Data are presented as mean ± standard deviation.

Demographic characteristics were compared by means of the Wilcoxon rank-sum and Fisher's exact tests. The Mantel-Haenszel $\chi^2$ test was used to compare clinical and bacteriologic outcomes with the 2 treatments. Duration of hospitalization for the 2 groups was compared with the Wilcoxon rank-sum test.

### RESULTS

Of the 93 women who entered the trial, 27 of 47 treated with ampicillin/sulbactam and 34 of 46 given cefoxitin were evaluable for clinical and bacteriologic efficacy (Table 1). In both treatment groups, patients not evaluable were primarily those lost to follow-up. The demographic characteristics evaluated were statistically similar between groups ($P \geqslant 0.34$ for all variables). Seven patients in the ampicillin/sulbactam group and 4 in the cefoxitin group had documented abdominal abscesses.

Reasons for exclusion from efficacy analyses (Table 2) were failure to receive the minimum number of doses (11 ampicillin/sulbactam vs. 3 cefoxitin), concomitant antibiotic therapy (3 vs. 4), administration of doxycycline outside the treatment window (3 vs. 0), positive results on test for human immunodeficiency virus (HIV) (1 vs. 3), failure to confirm the initial diagnosis of PID (1 vs. 2), and age <14 years (1 vs. 0). Concomitant antibiotics were administered due either to failure of study medication or to nursing error.

No significant difference in clinical efficacy was noted between the 2 treatments ($P = 0.67$) (Fig. 1). Of the 27 evaluable patients who received ampicillin/sulbactam, 67% (N = 18) were cured and 30% (N = 8) were improved; treatment failed in 4% (N = 1). Respective values for the 34 evaluable patients treated with cefoxitin were 68% (N = 23),

TABLE 2. Number of patients excluded from standard efficacy analysis

| Reason for non-evaluability | Ampicillin/sulbactam | Cefoxitin |
|---|---|---|
| Received fewer than the minimum number of total doses of study medication | 11 | 3 |
| Received concomitant antibiotic | 3 | 4 |
| Doxycycline administered outside of treatment window | 3 | 0 |
| Diagnosis of PID was not confirmed | 1 | 2 |
| Tested positive for HIV | 1 | 3 |
| Patient's age was below minimum age allowable for study participation | 1 | 0 |
| Total | 20 | 12 |

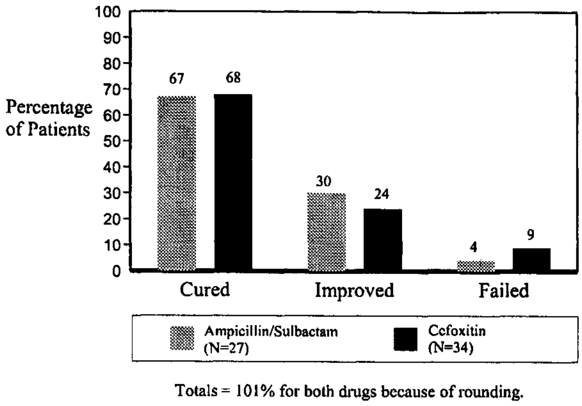

Fig. 1. Clinical responses to ampicillin/sulbactam and cefoxitin.

24% (N = 8), and 9% (N = 3). In both treatment groups, most patients who were clinically improved required antibiotic therapy that continued post-study. None required surgical intervention.

Primary pathogens isolated in each treatment group are detailed in Table 3. Overall, ampicillin/sulbactam eradicated a higher percentage of causative pathogens than cefoxitin, although the difference was not statistically significant ($P = 0.64$) (Fig. 2). Pathogens were eradicated in 70% (N = 19) of evaluable patients treated with ampicillin/sulbactam. Eradication followed by superinfection occurred in 7% (N = 2), persistence in 4% (N = 1), and 19% (N = 5) had an indeterminate response. Patients with indeterminate responses were primarily those for whom no adequate post-therapy culture was obtained or to whom other IV antibiotics were being administered at study entry. Values for cefoxitin-treated patients were 56% (N = 19), 9% (N = 3), 3% (N = 1), and 26% (N = 9). Two patients (6%) in this group experienced partial eradication of causative pathogens.

TABLE 3. Primary bacterial isolates in evaluable patients by treatment group

| | Ampicillin/sulbactam (N = 108 isolates) | Cefoxitin (N = 115 isolates) |
|---|---|---|
| *Neisseria gonorrhoeae* | 38 | 36 |
| *Mycoplasma hominis* | 36 | 36 |
| *Ureaplasma urealyticum* | 17 | 25 |
| *Chlamydia trachomatis* | 13 | 11 |
| Other | 4 | 7 |

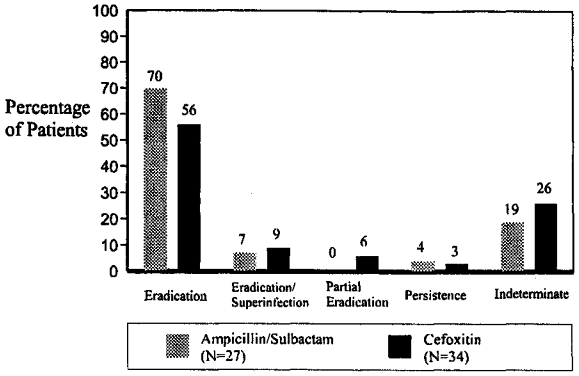

Fig. 2. Bacteriologic responses to ampicillin/sulbactam and cefoxitin.

Organisms responsible for superinfection were *M. hominis* and *C. trachomatis* in patients receiving ampicillin/sulbactam, and *N. gonorrhoeae* and *M. hominis* in patients treated with cefoxitin. Indeterminate responses were primarily due to the absence of post-therapy culture results and the administration of IV antibiotics prior to study entry.

In only 2 of the 13 patients with documented *C. trachomatis* infection in the ampicillin/sulbactam treatment group did infection persist post-treatment. Infection persisted post-treatment in none of the 11 patients in the cefoxitin treatment group with documented *C. trachomatis* infection.

The average (mean ± standard deviation) duration of hospitalization was 4.96 ± 0.94 days for the patients treated with ampicillin/sulbactam and 5 ± 1.73 days for the patients who received cefoxitin ($P = 0.85$). In both groups, duration of hospitalization was slightly longer (1–2 days) in most treatment failures.

All patients who received study medication were included in an intent-to-treat analysis.

In the intent-to-treat analysis, 19/47 (40%) of ampicillin/sulbactam-treated patients were cured, 8/47 (17%) improved, 1/47 (2%) failed, and 19/47 (40%) had indeterminate results. In the cefoxitin treatment group, 23/46 (50%) were cured, 8/46 (17%) improved, 3/46 (7%) failed, and 12/46 (26%) had indeterminate results. There was no statistically significant difference between treatment groups for clinical outcome ($P$ = 0.40, Mantel-Haenszel $\chi^2$ test).

Pathogens were eradicated in 20/47 (43%) of ampicillin/sulbactam-treated patients. Eradication followed by superinfection occurred in 2/47 (4%), partial eradication in 0/47 (0%), persistence in 1/47 (1%), and in 24/47 (51%), bacteriologic response was considered indeterminate. In the cefoxitin-treated group, eradication occurred in 19/46 (41%), eradication followed by superinfection in 3/46 (7%), partial eradication in 2/46 (4%), and persistence in 1/46 (2%). In 21/46 (46%), the bacteriologic response was considered indeterminate. There was no statistically significant difference between the treatment groups ($P$ = 0.67, Mantel-Haenszel $\chi^2$ test).

In the intent-to-treat analysis, the mean durations of hospitalization for the ampicillin/sulbactam and cefoxitin groups were 4.87 ± 1.28 and 4.89 ± 1.64 days, respectively ($P$ = 0.72, Wilcoxon rank-sum test).

Adverse events were reported in 56% (N = 25) of 47 patients who received ampicillin/sulbactam and 61% (N = 28) of 46 patients treated with cefoxitin. The most common events were vaginal yeast infection (28% of ampicillin/sulbactam-treated patients vs. 17% of cefoxitin-treated patients), constipation (11% vs. 7%), and nausea/vomiting (4% vs. 11%) (Table 4).

**TABLE 4. Most common clinical and laboratory adverse events (%)**

| | Ampicillin/sulbactam (N = 47) | Cefoxitin (N = 46) |
|---|---|---|
| Clinical | | |
| Vaginal yeast infection | 28 | 17 |
| Constipation | 11 | 7 |
| Nausea/vomiting | 4 | 11 |
| Abdominal pain | 2 | 2 |
| Itching at injection site | 2 | 2 |
| Headache | 2 | 2 |
| Diarrhea | 2 | 4 |
| Laboratory | | |
| Anemia | 6 | 7 |
| Neutropenia | 6 | 4 |
| Eosinophilia | 4 | 0 |
| Elevated SGOT | 4 | 0 |

### DISCUSSION

The results of this controlled clinical trial indicate that ampicillin/sulbactam is as safe and effective for the treatment of PID as cefoxitin. These findings are consistent with those from a number of other studies comparing ampicillin/sulbactam with either this cephamycin or combination therapies for PID. McGregor et al.[12] compared ampicillin/sulbactam with cefoxitin plus doxycycline in 103 evaluable patients with PID and noted no significant difference in clinical response rates. Hemsell et al.[9] compared ampicillin/sulbactam and cefoxitin in 117 patients with either uncomplicated or complicated acute PID. In this trial, the significantly greater efficacy of ampicillin/sulbactam resulted from the combination's activity in PID complicated by a pelvic inflammatory mass. Duration of therapy was also shorter for patients with complicated PID who received ampicillin/sulbactam than for those treated with cefoxitin. The bacteriologic efficacy of ampicillin/sulbactam was equivalent to that of cefoxitin for eradication of pathogens associated with PID.[10]

In comparison with other traditional combinations, ampicillin/sulbactam achieved clinical cure rates comparable to those with metronidazole plus gentamicin[15] and clindamycin plus gentamicin[16] in women with PID.

The excellent activity of ampicillin/sulbactam against *N. gonorrhoeae* and both gram-positive and gram-negative aerobes and anaerobes frequently involved in PID is well documented,[9,10,12] but like cefoxitin, ampicillin/sulbactam is less active against *C. trachomatis.* Although several investigations have suggested that β-lactam antibiotics may be effective in *C. trachomatis* infections,[17,18] Kosseim et al.[19] reported that ampicillin/sulbactam appeared to suppress rather than eradicate this pathogen. The investigators' recommendation—administration of doxycycline together with ampicillin/sulbactam for

*C. trachomatis*-associated PID—was adopted in the present study.

A recent meta-analysis evaluated the effectiveness of a large number of single-drug and combination therapies in women with PID.[20] Ampicillin/sulbactam, with the addition of doxycycline when appropriate, had clinical and bacteriologic efficacy comparable to that of clindamycin plus gentamicin, tobramycin, or amikacin; metronidazole plus doxycycline; either cefoxitin, cefotetan, or cefotaxime plus doxycycline; ciprofloxacin or ofloxacin; or the combination of amoxicillin, clavulanic acid, and doxycycline. Ampicillin/sulbactam was also less expensive than most of these regimens, including those involving cefoxitin.[20] This last conclusion is supported by results of a recent pharmacoeconomic analysis that directly compared the cost-effectiveness of these two agents. Conversion from cefoxitin to ampicillin/sulbactam at a university teaching hospital resulted in an initial annual savings of $30,000 to $50,000.[21]

In summary, PID, a common infection among sexually active women, may entail a significant risk of long-term sequelae, including chronic abdominal pain, ectopic pregnancy, and infertility.[22] Effective treatment of this disease requires broad antimicrobial coverage that traditionally has been achieved with relatively expensive combination therapy involving administration of as many as three agents with different dosage schedules.[23,24] The combination of the β-lactam ampicillin with the β-lactamase inhibitor sulbactam has demonstrated excellent clinical and bacteriologic efficacy in patients with PID, and is less expensive than many other therapies, including cefoxitin.

### REFERENCES

1. Rolfs RT, Galaid EI, Zaidi AA: Pelvic inflammatory disease: Trends in hospitalizations and office visits, 1979 through 1988. Am J Obstet Gynecol 166:983–990, 1992.
2. Dodson MG: Antibiotic regimens for treating acute pelvic inflammatory disease. J Reprod Med 39:285–296, 1994.
3. Peterson HB, Galaid EI, Zenilman JM: Pelvic inflammatory disease: Review of treatment options. Rev Infect Dis 12(Suppl 6):S656–S664, 1990.
4. King LA: Pelvic inflammatory disease: Its pathogenesis, diagnosis, and treatment. Postgrad Med 81:105–114, 1987.
5. Venezio FR, O'Keefe JP: Microbiologic considerations in the treatment of serious pelvic infections in women. J Reprod Med 33(Suppl):124–127, 1988.
6. Soper DE: Pelvic inflammatory disease. Infect Dis Clin North Am 8:821–840, 1994.
7. Crombleholme WR, Ohm-Smith M, Robbie MO, Dekay V, Sweet RL: Ampicillin/sulbactam versus metronidazole-gentamicin in the treatment of soft tissue pelvic infections. Am J Obstet Gynecol 156:507–512, 1987.
8. Dodson MG: Optimum therapy for acute pelvic inflammatory disease. Drugs 39:511–522, 1990.
9. Hemsell DL, Wendel GD Jr, Hemsell PG, Heard ML, Nobles BJ: Inpatient treatment for uncomplicated and complicated acute pelvic inflammatory disease: Ampicillin/sulbactam vs. cefoxitin. Infect Dis Obstet Gynecol 1:123–129, 1993.
10. Hemsell DL, Heard MC, Hemsell PG, Nobles BJ: Sulbactam/ampicillin versus cefoxitin for uncomplicated and complicated acute pelvic inflammatory disease. Drugs 35(Suppl 7):39–42, 1988.
11. Centers for Disease Control: Sexually transmitted diseases treatment guidelines 1982. MMWR 31(Suppl 2): 33S–60S, 1982.
12. McGregor JA, Crombleholme WR, Newton E, Sweet RL, Tuomala R, Gibbs RS: Randomized comparison of ampicillin-sulbactam to cefoxitin and doxycycline or clindamycin and gentamicin in the treatment of pelvic inflammatory disease or endometritis. Obstet Gynecol 83:998–1004, 1994.
13. Murray PR, Cantrell HF, Lankford RB: Multicenter evaluation of the in vitro activity of piperacillin-tazobactam compared with eleven selected beta-lactam antibiotics and ciprofloxacin against more than 42,000 aerobic gram-positive and gram-negative bacteria. In Vitro Susceptibility Surveillance Group (abstract). Diagn Microbiol Infect Dis 19:111–120, 1994.
14. Benson JM, Nahata MC: Sulbactam/ampicillin, a new beta-lactamase inhibitor/beta-lactam antibiotic combination (abstract). Drug Intell Clin Pharm 22:534–541, 1988.
15. Crombleholme W, Landers D, Ohm-Smith M, et al.: Sulbactam/ampicillin versus metronidazole/gentamicin in the treatment of severe pelvic infections. Drugs 31(Suppl 2):11–13, 1986.
16. Gunning J: A comparison of parenteral sulbactam/ampicillin versus clindamycin/gentamicin in the treatment of pelvic inflammatory disease. Drugs 31(Suppl 2):14–17, 1986.
17. Crombleholme WR, Schachter J, Grossman M, Landers DV, Sweet RL: Amoxicillin therapy for *Chlamydia trachomatis* in pregnancy. Obstet Gynecol 75:752–756, 1990.
18. Bowie WR, Alexander ER, Holmes KK: Eradication of *Chlamydia trachomatis* from the urethra of men with nongonococcal urethritis by treatment with amoxicillin (abstract). Sex Transm Dis 8:79–81, 1981.
19. Kosseim M, Ronald A, Plummer FA, D'Costa L, Brunham RC: Treatment of acute pelvic inflammatory disease in the ambulatory setting: Trial of cefoxitin and doxycycline versus ampicillin-sulbactam. Antimicrob Agents Chemother 35:1651–1656, 1991.

20. Walker CK, Kahn JG, Washington AE, Peterson HB, Sweet RL: Pelvic inflammatory disease: Meta-analysis of antimicrobial regimen efficacy. J Infect Dis 168:969–978, 1993.
21. Kitrenos JG, Rotella DL, Nakasato S: Conversion of treatment from cefoxitin to ampicillin/sulbactam: Experience in a university teaching hospital. Adv Ther 12: 30–43, 1995.
22. Safrin S, Schachter J, Dahrouge D, Sweet RL: Long-term sequelae of acute pelvic inflammatory disease: A retrospective cohort study. Am J Obstet Gynecol 166: 1300–1305, 1992.
23. Peterson HB, Galaid EI, Cates W Jr: Pelvic inflammatory disease. Med Clin North Am 74:1603–1615, 1990.
24. Uri FI, Sartawi SA, Dajani YF, Masoud AA, Barakat HF: Amoxycillin/clavulanic acid (augmentin) compared with triple drug therapy for pelvic inflammatory disease. Int J Gynecol Obstet 38:41–43, 1992.
